# Older adult fall prevention practices among primary care providers at accountable care organizations: A pilot study

**DOI:** 10.1371/journal.pone.0205279

**Published:** 2018-10-11

**Authors:** Jonathan Howland, Holly Hackman, Alyssa Taylor, Kathleen O’Hara, James Liu, John Brusch

**Affiliations:** 1 Department of Emergency Medicine, Boston University Medical Center and Boston University School of Medicine, Boston, Massachusetts, United States of America; 2 Boston Medical Center Injury Prevention Center, Boston, Massachusetts, United States of America; 3 Cambridge Health Alliance, Cambridge, Massachusetts, United States of America; University of Illinois at Urbana-Champaign, UNITED STATES

## Abstract

**Background:**

Falls are a serious and common problem among older adults. Low-tech, inexpensive, community-based fall prevention programs have been shown to be both effective and cost effective, however, these programs are not well-integrated into clinical practice.

**Research design:**

We surveyed primary care providers at a convenience sample of two accountable care organizations in Massachusetts to assess their beliefs, attitudes, knowledge, and practices relative to fall risk assessment and intervention for their older patients.

**Results:**

Response rate was 71%. Providers’ beliefs about the efficacy of fall risk assessment and intervention were mixed. Eighty-seven percent believed that they could be effective in reducing fall risk among their older adult patients. Ninety-six percent believed that all older adults should be assessed for fall risk; and, 85% believed that this assessment would identify fall risk factors that could be modified. Nonetheless, only 52% believed that they had the expertise to conduct fall risk assessment and only 68% believed that assessing older adult patients for fall risk was the prevailing standard of practice among their peer providers. Although most providers believed it likely that an evidence-based program could reduce fall risk among their patients, only 14% were aware of the Centers for Disease Control and Prevention’s fall risk assessment algorithm (STEADI Toolkit), and only 15% were familiar with Matter of Balance, the most widely disseminated community fall risk prevention program in Massachusetts.

**Discussion:**

New strategies that more directly target providers are needed to accelerate integration of fall risk assessment and intervention into primary care practice.

## Introduction

Falls among older adults are common. Each year, a quarter of those 65 years of age or older fall. These falls can result in debilitating, sometimes fatal, injuries and affect psychosocial status and quality of life. Among older adults, falls are the leading cause of fatal and non-fatal injuries [1]. In 2015, 2.5 million older adults in the U.S. were treated in emergency departments (EDs) for non-fatal fall-related injuries and more than 734,000 of these patients were hospitalized [2]. In that year, the direct medical costs for older adult falls exceeded $50 billion [3]. Even when falls do not require medical attention, the experience can result in fear of falling, which can be psychologically disabling [4] and lead to future falls through physical deconditioning [5,6].

Over recent decades, community-based fall prevention interventions have been developed and subjected to randomized trials [7]. These low cost, low-tech programs can result in 25–30% reductions in falls one-year post-program [7]. These programs, however, are not well-integrated into clinical practice and are most often offered by non-medical public and private organizations that serve older adults. Because these programs are typically marketed directly to the public, rather than through referrals from healthcare providers, they may not serve many older adults with the most to benefit from participation.

Recent studies have also shown community-based fall prevention programs to be cost-effective. The Centers for Medicare and Medicaid Services [8] conducted a retrospective cohort study evaluating Matter of Balance (MOB), a program developed to reduce fear of falling and increase mobility in older adults [9,10]. Compared to matched controls, older adults who had participated in the MOB program had significantly lower total health care costs during the post-participation year [8]. Another study estimated the net benefit and return on investment (ROI) of three evidence-based fall prevention programs [11]. Otago, a program targeting frail older adults that is delivered in the home by a physical therapist or other healthcare provider [12], had a one-year net benefit of $121.85 and a ROI of 36% for each dollar invested. Tai Chi: Moving for Better Balance, a group program for enhancing strength and balance [13], had a one-year net benefit of $529.86 and a ROI of 509% for each dollar invested. Stepping On, a program combining community-based group sessions with follow-up home visits by a healthcare provider [14], had a 14-month net benefit of $134.37 and a ROI of 64% for each dollar invested [11]. In a separate study, Howland et al. estimated a ROI of 144%, if all older adults presenting with a fall injury at Massachusetts EDs were referred to MOB and 50% complied and completed the program [15].

In addition to the development and evaluation of interventions to reduce fall risk, new risk assessment algorithms have been developed and promoted. Most notable among these is the STEADI (Stop Elderly Accidents, Deaths and Injuries) Toolkit [16], which was developed by the Centers for Disease Control and Prevention (CDC) for use in clinical settings. The STEADI algorithm outlines a standardized approach for healthcare providers to conduct fall risk screening, assessment, and intervention for older adults. Among the assessment tools recommended by STEADI are the Timed Up and Go Test [16], a test for mobility and recommended for all patients who screen positive to the fall risk screening questions, and the 4-Stage Balance Test, an optional test for assessment of balance.

There have been relatively few studies of provider practices for fall risk assessment and intervention. Wegner et al. queried a sample or community-dwelling older adults enrolled in two managed care organizations who had received care in 1998–1999 and found that most were not asked about their fall history [17]. Jones et al. surveyed a random sample of Colorado primary care physicians about older adult fall prevention practices [18]. Only 8% of respondents reported fall prevention practices based on guidelines from recognized organizations; lack of time, more pressing medical problems and lack of educational materials were the most frequently cited barriers to fall risk assessment [18]. Among 38 healthcare providers from 11 New York state practices, Smith et al. found that less than 40% asked most or all their older adult patients if they had fallen in the last year; less than 20% referred their older patients to community-based fall prevention programs; and, less than 16% conducted standardized functional assessment with their older patients at least once a year [19]. Burns et al. analyzed data on fall prevention recommendations to older adult patients among 1210 US primary care providers who participated in the 2014 DocStyles survey [20]. These investigators found significant practice differences by provider type, suggesting the absence of provider consensus on fall prevention guidelines [20].

For the present study, we surveyed a convenience sample of primary care providers to assess their beliefs, attitudes, knowledge, and practices relative to fall risk assessment and intervention for older adult patients. The purpose was to gauge the extent to which providers were assessing fall risk in older adult patients and referring these patients to evidence-based fall prevention interventions.

## Materials and methods

### Derivation of questionnaire

Survey questions were derived from several sources, including, replicated or modified questions from the National Council on Aging’s Evaluation Guidelines for Falls Prevention Coalitions [21], the CDC’s Clinician Baseline Questionnaire, which was developed for evaluating an on-line physician training program for the STEADI Toolkit [16], American Geriatric Society and British Geriatric Society’s (AGE/BGS) best practice guidelines [22], and a study by Nyrop et al. [23]. Other questions were developed specifically for the present study.

Questions reflected four dimensions relative to older adult fall risk assessment and intervention: provider beliefs, knowledge, attitudes, and clinical practices. Questions about beliefs aimed to determine the extent to which providers endorsed that they could effectively mitigate their older adult patients’ risk for falling. Knowledge questions asked about providers’ expertise relative to fall risk assessment and intervention; their awareness of assessment tools; and, their awareness of several evidenced-based community programs for preventing falls and reducing fear of falling. Attitude questions focused on adequacy of time and reimbursement for assessing older adult fall risk. Practice questions asked about the frequency with which providers conducted various fall assessment and intervention practices. We also collected information on respondents’ demographics and the characteristics of their patients. Table 1 shows all the questions included in the survey and their derivations.

**Table 1:** Survey questions and sources.

**Beliefs**	
I can do things for my independently-living older adult patients to reduce their risk of falling.	NCOA Evaluation Guidelines for Fall Prevention Coalitions
All patients ages 65 and older should be assessed for falls risk.	Nyrop Physician Perspective on Fall Prevention in Assisted Living (modified)
A falls risk assessment will uncover risks that can be modified	Nyrop Physician Perspective on Fall Prevention in Assisted Living (modified)
An evidence-based community falls prevention program can reduce the risk for falls among older adult patients identified as high risk.	Unique to project
I (or my office staff) have the expertise to do fall risk assessments of my patients ages 65 and older.	Unique to project
It is the prevailing community standard among my professional peers to assess the risk for falls in older adult patients.	Unique to project
**Knowledge**	
Are you aware of the falls risk assessment toolkit developed by the Centers for Disease Control and Prevention called STEADI?	Unique to project
Are you familiar with any of the following evidence-based community fall prevention programs?	
Matter of Balance	Unique to project
Tai Chi: Moving for Better Balance	
The Otago Exercise Program	
**Attitudes**	
I (or my office staff) have the time to do fall risk assessments of my patients ages 65 and older.	CDC STEADI Toolkit: Clinician Baseline Questionnaire (modified)
I am adequately reimbursed for doing fall risk assessments of my patients ages 65 and older.	Unique to project
**Practices**	
Do you (or your office staff) routinely use the STEADI Toolkit to assess your older adult patients for fall risk?	Unique to project
Over the past 12 months, for approximately what percent of your independently-living patients ages 65 and older did you (or your office staff) …	
Conduct a falls history?	AGS/BGS Clinical Guideline (2010) (modified)
Review medications for falls risk?	
Assess visual acuity?	
Conduct the Timed Up and Go (TUG) test?	
Conduct the 4-Stage Balance test?	
Educate about specific fall risk factors?	
Screen for Vitamin D deficiency?	
Refer to evidence-based community fall prevention programs?	CDC STEADI Toolkit: Clinician Baseline Questionnaire (modified)
**Respondent Characteristics**	
What type of medical degree do you have?	
What is your gender?	
How many years ago did you complete your medical degree	
**Site Characteristics**	
Approximately what percent of your office visits are patients ages 65 and older?	
Approximately what percent of your patients ages 65 and older would be considered low income ($30,000/year or less)?	
Approximately what percent of your older adult patients fall into the following race/ethnicity categories: White (non-Hispanic); Black (non-Hispanic); Hispanic/Latino; Asian/pacific Islander; and, Other?	
Approximately what percent of your older adult patients use a primary language other than English?	

### Sites and survey administration

Accountable care organizations (ACOs) are integrated healthcare provider organizations that include physicians, hospitals, and other providers to offer coordinated patient care to enhance quality of care and contain healthcare costs. ACOs contract with payers using “alternative payment methods” under which the ACO is responsible for the health care and health outcomes of attributed patients. If budget and quality goals are met, the ACO shares in the cost saving; if these goals are not met, the ACO bears a portion of the losses. In 2017, Massachusetts established the nation’s first standards for (ACOs) and 17 ACOs were certified in the state that year.

A convenience sample of five of the 17 ACOs was selected based on proximity to investigators (to facilitate in-person meetings) and the large size of their patient populations. The executive director of each organization was sent an information package, including a copy of the questionnaire with a cover letter, signed by the Commissioner of the Massachusetts Department of Public Health and by the state’s Secretary of the Executive Office of Elder Affairs. The cover letter introduced the study, requested a response about willingness to consider participation, and requested designation of a contact person within the organization with whom the study staff could discuss survey aims, content, and implementation. Three organizations responded, of which two agreed to participate (P1 and P2).

P1 is a vertically integrated ACO that serves urban communities in Eastern Massachusetts and offers inpatient services, primary care, specialty care, mental health, and substance abuse treatment. P2 is an ACO that provides primary and specialty care services to urban and suburban communities in Central Massachusetts and Boston MetroWest.

The investigators worked with the designated contact person to distribute the survey. The organizations identified eligible clinicians to whom the survey was administered. Physicians engaged in adult primary care and who care for older adult patients were the target of the survey, however, in some cases, nurse practitioners and physician assistants were included in the distribution. At P1, the survey was completed on-line and anonymously using the survey tool Qualtrics. Three reminder follow-ups were subsequently sent to non-respondents. At P2, the contact person distributed hard copies of the survey, which were returned anonymously by mail to the study staff.

The survey was administered in May, 2016 and data collection was continued through August 2016.

### Data analyses

For questions that had a response consisting of a six-point agreement/disagreement scale, we dichotomized responses 1–3 as disagreement and 4–6 as agreement. Chi-square and Student’s t-test were used to compare categorical and continuous P1 and P2 responses; significance was set at alpha = .05. Data were analyzed using Microsoft Excel and SAS v9.4.

### Human subjects

This study was reviewed by the Institutional Review Board at Boston Medical Center.

## Results

### Response rates

In total, 136 surveys were distributed (90 P1 providers; 46 P2 providers). Overall, 97 of 136 (71%) of targeted providers responded to the survey (73% of P1; 67% of P2; p = .47).

### Respondent characteristics

Ninety-three percent (89% of P1; 94% of P2; p = .76) of respondents were MDs. All those who responded “Other” were physician assistants, nurse practitioners, or did not specify.

Respondents at P1 and P2 did not differ significantly with respect to gender but did differ significantly with respect to years since graduation from medical school and specialty (Table 2).

**Table 2:** Respondent characteristics.

Characteristics	P1	P2	P Value
% Male	33.90%	51.60%	0.11
Years since Graduation	Mean = 15.2	Mean = 23.1	0.01
	SD = 12.7	SD = 12.3	
% MD	89%	94%	0.76
Geriatrics	4.8% (3)	12.1% (4)	0.045
Internal Medicine	51.60%	69.70%	
Family Practice	33.80%	18.20%	
Other	9.70%	0.00%	

### Site characteristics

P1 and P2 differed with respect to patient characteristics: percent of office visits by patients who were ≥65 years of age (25.6% vs. 43.2%; p<0.01); at least 50% of older adult patients were low income (80% vs. 44%; p<0.01); proportion of older adult patients who were minority (59% vs. 35%; p<0.01); and, percent of patients whose primary language was non-English (47% vs. 23%; p<0.01).

### Respondent beliefs

Eighty-seven percent (83% of P1; 94% of P2; p = 0.16) agreed that they could do things to prevent their independently-living patients from falling. Ninety-six percent (98% of P1; 90% of P2; p = 0.08) agreed that all patients ages 65 and older should be assessed for falls risk. Eighty-five percent (83% of P1; 90% of P2; p = 0.34) agreed that a fall risk assessment will uncover factors that can be modified. Ninety-four percent (93% of P1; 94% of P2; p = 0.94) endorsed as likely that evidence-based community fall prevention programs can reduce fall risk among high risk older adult patients. Fifty-two percent (53% of P1; 50% of P2; p = 0.76) agreed that they had the expertise to perform fall risk assessments. Sixty-eight percent (73% of P1; 57% of P2; p = 0.12) agreed that it is the prevailing standard among professional peers to assess fall risk for of their older adult patients.

### Respondent knowledge

Fourteen percent of respondents (14% of P1; 14% of P2; p = 0.95) were aware of the STEADI falls risk assessment toolkit [16]. Fifteen percent (19% of P1; 7% of P2; p = 0.20) were familiar with MOB [9,10]; 43% (40% of P1; 50% of P2; p = 0.49) were familiar with Tai Chi: Moving for Better Balance [13]; and, less than 1% of respondents (2% of P1; 0% of P2) were familiar with Otago [12].

### Respondent attitudes

Fifty percent of respondents (53% of P1; 43% of P2; p = 0.36) agreed that they had the time to perform fall risk assessment of older adult patients. Twenty-four percent of respondents (27% of P1; 18% of P2; p = 0.33) agreed that they were adequately reimbursed for performing fall risk assessments for their independently-living older adult patients.

### Respondent practices

Of those who reported awareness of the STEADI Toolkit [16] (N = 8), 50% (63% of P1; 25% of P2 respondents; p = 0.30) indicated that they (or their office staff) routinely used the STEADI Toolkit to assess their independently-living older adult patients for fall risk.

With respect to conducting assessments recommended by the AGS/BGS Guidelines [22], P1 and P2 did not differ significantly on any of the component parts. On average, they reported assessing at least 50% of their older adult patients during the past year for falls history (59.8%), medication regimen (61.5%), and vitamin D deficiency (50.9%). Other assessments were conducted for less than 50% of older adult patients during the past year: vision (38.8%); Timed Up and Go Test (TUG) (20.6%); 4-Stage Balance Test (3.6%). With respect to interventions recommended by the AGS/BGS providers counseled an average of 47% of older adult patients during the past year and made referrals to fall prevention programs for 9.1%. See Table 3.

**Table 3:** Fall assessment & intervention practices.

Questions	P1		P2		p-value	Mean %
	Mean % (SD)	n	Mean % (SD)	n		
Conduct falls history	57.8 (26.6)	58	63.8 (33.3)	28	0.38	59.8
Review medications	57.8 (29.3)	58	68.1 (35.5)	28	0.12	61.5
Assess vision	35.6 (26.0)	57	45.1 (30.0)	28	0.14	38.8
Conduct TUG	19.1 (26.9)	57	23.8 (31.2)	28	0.48	20.6
Conduct 4-Stage balance test	2.6 (13.6)	57	5.7 (14.7)	27	0.34	3.6
Educate on fall risk	46.3 (29.7)	58	48.6 (28.6)	28	0.70	47.0
Screen for Vitamin D deficiency	50.3 (29.2)	58	52.3 (30.1)	28	0.78	50.9
Refer to evidence-based programs	7.1 (13.0)	57	13.4 (23.3)	26	0.20	9.1

## Discussion

Our findings suggest that despite their efficacy and potential for cost saving, fall-risk assessment tools and community-based fall prevention programs are not well-integrated into clinical practice. Because fall prevention programs are often offered by public and private organizations that serve older adults, most are marketed directly to the public and participants are self-selected. This model presents several problems. First, people who elect to go directly to community-based programs, without seeing a clinician about their risk for falls, may have underlying health conditions that increase fall risk and need medical management. As a result, some older adults may not get, or may delay, the medical attention they need to address fall-related medical problems.

Second, low self-efficacy with respect to fall prevention is likely a risk for falling, to the degree that it limits individuals’ fall prevention mindfulness and associated activities. Those who elect to participate in a community fall prevention program already demonstrate some level of control over their risk for falling. In other words, the act of participating in fall prevention activities indicates some measure of fall self-efficacy prior to program enrollment. But patients who do not elect to participate, because they do not believe that fall risk can be modified, may be those with the most to benefit from fall prevention programs. Thus, many older adults with the greatest needs are not accessing the programs but might be persuaded to do so by their healthcare provider.

Third, for community-based fall prevention programs to have population-level impact, they must be broadly disseminated and engage a substantial portion of the older adult population. Large scale participation by older adults in community-based fall prevention programs will likely not occur unless individuals are referred to these programs by their physicians within the context of clinical care.

Although Massachusetts has been a leader in fall prevention initiatives, our findings indicate that further effort is required to increase integration of evidence-based fall prevention assessment algorithms and community fall prevention programs into primary care. In a recent study of fall prevention activities undertaken by older adults (n = 87) 60 days post-discharge from an urban Massachusetts emergency department, only 37% had spoken to their healthcare provider about fall prevention, 22% had spoken to their provider about medication risk for falls, 15% had spoken to their provider about their vision, 2% had attempted to contact a community-based falls prevention program, and none had participated in a falls prevention program [24].

New strategies that more directly target providers are needed to accelerate integration of fall risk assessment and intervention into primary care practice. For example, initiatives could be implemented to enhance education and training about older adult falls for medical students, and other relevant providers, at health provider educational institutions throughout the state. Similarly, continuing medical education on fall prevention could be made a requirement for initial licensure and renewal for relevant Massachusetts healthcare providers. A state or private agency could create and maintain a website that listed the time, place, and sponsor of community-based fall prevention programs, so that older adults and their healthcare providers could locate these programs for referral. Insurance coverage for community-based fall prevention programs by private and public third-party payers could do much to stimulate provider referrals. In the absence of reimbursement, however, ACOs might consider offering or sponsoring fall prevention, and other chronic disease self-management programs, to reduce health care costs among their attributed patients.

The investigators acknowledge several limitations of this study. First, the study used a convenience sample that included only two of 17 Massachusetts ACOs, and therefore generalizability (external validity) of findings to all Massachusetts ACOs or primary care providers cannot be made. Moreover, because ACOs have financial incentives relative to quality of care and cost containment, it is possible that primary care providers at ACOs are more apt that those at other provider organizations to practice preventive medicine. We invited five group practices to participate in this study. One never responded, two responded, but subsequently ceased communicating about the study, and two participated. It is possible that the self-selection of the two out of five organizations we approached could have biased findings if, for example, willingness to participate was associated with better fall risk assessment practices. We could have opted for drawing a sample from the Massachusetts physician licensure registry, but this approach has yielded poor response rates in the past. Thus, the methodological dilemma was a choice between a valid sampling procedure that risked a small response rate versus a convenience sample, of limited generalizability, that yielded acceptable response rates and thus valid data for participants. We chose the second option.

Despite the limitation on generalizability, it is noteworthy that in most respects, the two practices surveyed were very similar with respect to knowledge, beliefs, attitudes, and practices, with few statistically significant differences. This suggests that findings may apply to other primary care providers in the state because most findings were consistent across the participating practices.

Nonetheless, even if there were reason to believe that our findings might be generalizable to most Massachusetts primary care providers, our sample included no other state. In areas of overlap, however, our results were not dissimilar from those of other recent studies of provider practices relative to older adult fall prevention [17–20].

Second, as with any survey, responses can be biased by social desirability, the tendency of respondents to distort answers towards what they perceive to be normative. Many of our findings, however, remain important, even if they are inflated towards socially desirable answers. For example, even if some respondents indicated that they were aware of the STEADI Toolkit, when they were not aware, the finding that only 14% said they were aware remains a small proportion.

Third, our analyses of provider practices did not account for differences in patient case mix across providers or organizations.

Forth, while we asked providers if they referred their older adult patients to community-based fall prevention programs, we did not ask about fall prevention referrals to other providers, such as physical or occupational therapists, or general exercise programs such as those offered by YMCAs or Councils on Aging. This omission may have resulted in a failure to develop a complete picture of providers’ fall prevention practices for their older adult patients.

## Supporting information

S1 Table Primary care provider raw survey data. (XLSX)
